# Association Between Serum Magnesium and Muscle Mass in People With Type 2 Diabetes Mellitus

**DOI:** 10.1155/ije/9939748

**Published:** 2025-11-02

**Authors:** Lili Pei, Qiao Yang, Yuantao Liu, Wenchao Hu

**Affiliations:** ^1^Department of Endocrinology, Qilu Hospital (Qingdao), Cheeloo College of Medicine, Shandong University, Qingdao, China; ^2^Department of Cosmetic Medicine, Eighth People's Hospital of Qingdao, Qingdao, China

**Keywords:** low muscle mass, magnesium, sarcopenia, skeletal muscle index, type 2 diabetes mellitus

## Abstract

**Introduction:**

Muscle function and strength are related to magnesium (Mg). The risk of low muscle mass in type 2 diabetes mellitus (T2DM) is higher compared to healthy individuals. This study aimed to evaluate the association between serum Mg and low muscle mass in people with T2DM.

**Methods:**

This study included 1074 inpatients with T2DM with measured skeletal muscle index (SMI) and serum Mg concentrations, along with collected clinical characteristics. SMI was measured using dual-energy X-ray absorptiometry. Logistic regression analysis and linear regression analysis were employed to examine the associations between serum Mg concentrations and low muscle mass or SMI, respectively.

**Results:**

The prevalence of low muscle mass was 20.28% in males and 14.20% in females. Serum Mg concentration was significantly higher in the low muscle mass group compared to the normal muscle mass group. Furthermore, among female patients with T2DM, a negative correlation was observed between Mg levels and SMI. Multivariate logistic regression analysis revealed that high Mg levels were significantly associated with an increased risk of low muscle mass. Specifically, in females, higher serum Mg levels significantly increased the risk of low muscle mass. The odds ratios (95% confidence intervals) across increasing tertiles (T1 to T3) of serum Mg were 1.00 (reference), 1.321 (95% CI, 0.626–2.790), and 2.071 (95% CI, 1.011–4.243), respectively, with a significant trend (*p* for trend = 0.039).

**Conclusion:**

Low muscle mass in T2DM patients is associated with serum Mg levels. Notably, among female patients, higher serum Mg concentrations showed a significant linear trend and were negatively correlated with low muscle mass.

## 1. Background

Sarcopenia is age-related muscle atrophy characterized by a widespread decline in skeletal muscle mass, strength, and physical function [1]. It is related to a higher risk of morbidity, fractures, physical disability, and a lower quality of life [2]. A systematic review has reported that the prevalence of sarcopenia in community-dwelling Chinese older adults aged over 65 years was 17.4% [3]. Another study showed that sarcopenia prevalence among Chinese men aged 70 and above is 12.3%, while the rate for Chinese women is 7.6% [4]. Sarcopenia and its related comorbidities are becoming a serious health challenge for China's rapidly aging population [5]. The onset and progression of sarcopenia involve multiple factors, including old age, male gender, osteoporosis, physical inactivity, and malnutrition [6, 7]. Type 2 diabetes mellitus (T2DM) is a common metabolic disorder characterized by hyperglycemia, insulin resistance, insulin deficiency, or both. During the development of T2DM and the process of drug treatment, muscle mass loss is a noteworthy complication, especially for elderly patients [8]. The incidence of sarcopenia was significantly higher in T2DM patients than in healthy controls (14.8% vs. 11.2%). People with T2DM exhibited considerably increased risks of sarcopenia [7]. It is important to recognize and prevent sarcopenia in patients with T2DM.

Magnesium (Mg), an important mineral, is intimately related to several facets of skeletal muscle function, such as protein synthesis, energy production, and muscular contraction [9, 10]. Skeletal muscle is the major store of Mg. Dietary Mg intake is positively associated with skeletal muscle mass, and inadequate Mg intake results in the loss of muscle mass [11, 12]. However, studies that have explored the association between serum Mg status and skeletal muscle mass in people with T2DM are limited. The present study aimed to investigate whether serum Mg is associated with low muscle mass in patients with T2DM.

## 2. Methods

### 2.1. Patients

In this cross-sectional study, 1074 inpatients with T2DM were enrolled between September 2017 and September 2019 in the Qilu Hospital's endocrinology department in Qingdao. The subjects were adults with T2DM who had undergone dual-energy X-ray absorptiometry (DXA). Patients with a history of stroke, severe hip or knee osteoarthritis, malignancy, infectious illnesses, or pregnancy were not included. This study was approved by the hospital ethics board and carried out in compliance with the Declaration of Helsinki. All participants have taken written informed consent.

### 2.2. Definition of T2DM and Low Muscle Mass

T2DM was diagnosed according to the 1999 World Health Organization (WHO) criteria [13], with a fasting plasma glucose (FPG) level of ≥ 7.0 mmol/L and/or a 2 h postprandial plasma glucose level of ≥ 11.1 mmol/L. The appendicular skeletal muscle mass in kilograms divided by the square of the body height was the skeletal muscle index (SMI), which was determined using DXA (Hologic Discovery A, Waltham, MA, USA). SMI of less than 7.0 kg/m^2^ for male respondents or 5.4 kg/m^2^ for female subjects was considered low muscle mass [1].

### 2.3. Data Collection

Anthropometric (height, weight, and blood pressure), metabolic, and demographic (age and sex) factors were measured. Data on the duration of diabetes, medication regimen, and smoking/alcohol history were obtained from medical records. Body mass index (BMI) was calculated as weight (kg) divided by height squared (m^2^). Fasting blood samples (8–12 h overnight) were collected from all participants. FPG, total cholesterol (TC), triglyceride (TG), high-density lipoprotein cholesterol (HDL), low-density lipoprotein cholesterol (LDL), glycosylated hemoglobin (HbA1c), serum creatinine (Scr), blood urea nitrogen (BUN), calcium (Ca), phosphorus (P), and serum Mg were measured using an automatic biochemistry analyzer (Hitachi 7170, Hitachi; Tokyo, Japan). Serum Mg levels were categorized into tertiles, from the lowest to the highest (T1 to T3). Gender-specific cutoffs were applied: Males: T1 (< 1.96 mg/dL), T2 (1.96–2.11 mg/dL), T3 (≥ 2.11 mg/dL); Females: T1 (< 1.96 mg/dL), T2 (1.96–2.12 mg/dL), T3 (≥ 2.12 mg/dL).

### 2.4. Statistical Analysis

All statistical analyses were conducted using IBM's SPSS version 22.0 software (Chicago, IL). The means ± SDs are used to represent continuous variables. To find out how the characteristics of T2DM patients with and without reduced muscle mass differed, unpaired *t* tests were used. To find relationships between Mg, SMI, and other clinical traits, simple and multiple linear regression analyses were conducted. The risk variables for low muscle mass were identified through the use of univariate and multivariate logistic regression analysis. Because Mg was categorized into tertiles based on continuous data, a trend test could be used to investigate any associations with low muscle mass. To ascertain whether there is a linear relationship between Mg and low muscle mass, restricted cubic spline (RCS) analysis was performed. A *p* value < 0.05 was considered to indicate statistical significance.

## 3. Results

### 3.1. Differences Between Subjects With and Without Low Muscle Mass

The characteristics of the 1074 participants with T2DM (567 male, 507 female) are presented in Table 1. The overall prevalence of low muscle mass was 17.41%. Specifically, low muscle mass was identified in 115 males (20.28%) and 72 females (14.20%). Compared to males with normal muscle mass, those with low muscle mass were significantly older and had higher serum Mg, HDL-C, and HbA1c levels and a higher percentage of sulfonylurea and DPP-IV inhibitor treatment, as well as lower BMI, SMI, DBP, P, and TG levels. Similarly, females with low muscle mass were significantly older than those with normal muscle mass and demonstrated higher serum Mg levels, as well as lower BMI, SMI, TG levels, and percentage of metformin treatment.

### 3.2. Associations Between the SMI and Clinical Characteristics

As shown in Table 2, univariable linear regression analysis revealed significant associations between SMI and the following variables in males: age, Mg, SBP, DBP, duration of T2DM, TG, HDL-C, Ca, P, BUN, sulfonylureas, and DPP-IV inhibitor treatment. Subsequent multivariable linear regression analysis identified age, SBP, DBP, TG, HDL-C, and DPP-IV inhibitor treatment as factors independently associated with SMI in males. As displayed in Table 3, age, Mg, DBP, HDL-C, and P were correlated with SMI in female subjects after univariable linear regression analysis. In the adjusted multivariable linear regression model, age, Mg, DBP, and P remained independently associated with SMI in females.

### 3.3. Associations Between Mg and Other Clinical Characteristics

Univariable linear regression analysis revealed that Mg was correlated with age, BMI, TC, FPG, HbA1c, Scr, BUN, and metformin treatment in males. Multivariable linear regression found that age, TC, FPG, Scr, and metformin treatment remained correlated with Mg (Table 4). Among females, univariable linear regression analysis demonstrated that Mg was associated with age, BMI, duration of T2DM, TG, FPG, HbA1c, Scr, smoking, and drinking, treatment with metformin, acarbose, sulfonylureas, and insulin. In the adjusted multivariable model, age, BMI, FPG, Scr, and treatment with metformin, sulfonylureas, and insulin remained associated with Mg (Table 5).

### 3.4. Association Between Mg and Low Muscle Mass

**Figure 1:**
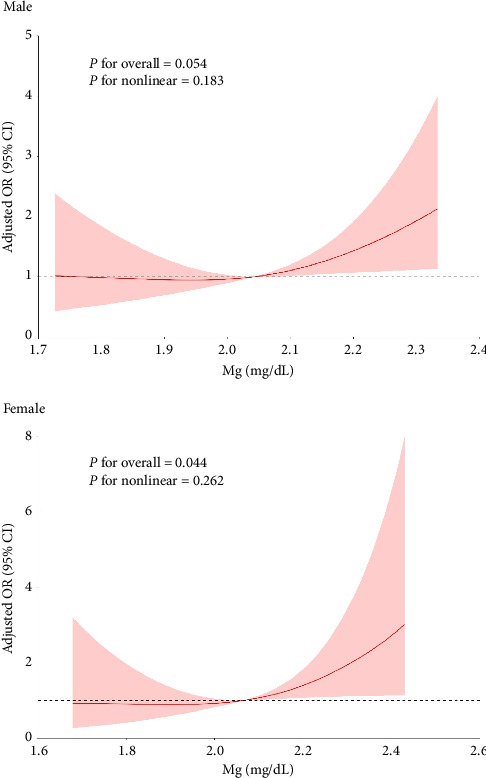
Restricted cubic spline plots on the relationship between Mg and low muscle mass caption: The red line in the figure represents OR, and pink line represents the 95% CI.

The association between serum Mg concentrations and low muscle mass is presented in Table 6. The prevalence of low muscle mass across increasing serum Mg tertiles was 18.71% (T1), 17.28% (T2), and 24.39% (T3) in males (Table 6). For females, the prevalence increased across tertiles: 8.39% (T1), 12.87% (T2), and 20.44% (T3) (Table 7). In Figure 1, RCS analysis revealed no significant nonlinear relationship between serum Mg levels and low muscle mass (both *p* for nonlinear > 0.05). After adjusting for age, duration of T2DM, SBP, DBP, TC, TG, HDL-C, LDL-C, FPG, BUN, Scr, P, and Ca (Model 3), high serum Mg levels in females were significantly associated with an increased risk of low muscle mass. The odds ratio (95% confidence intervals) compared to T1 (reference) were T2: 1.321 (95% CI, 0.626–2.790), T3: 2.071 (95% CI, 1.011–4.243) (*p* for trend = 0.039). High Mg levels significantly increased the risk of low muscle mass for females. In contrast, no significant association or linear trend was observed between Mg and low muscle mass in males (*p* for trend = 0.799).

## 4. Discussion

This study demonstrated that low muscle mass in T2DM patients was associated with serum Mg levels. Notably, among female patients, a significant linear trend and negative correlation were observed between serum Mg concentrations and muscle mass.

Sarcopenia is characterized by low muscle mass and low muscle function [14]. The prevalence of sarcopenia shows considerable heterogeneity. According to the European Consensus on Sarcopenia, the prevalence of sarcopenia was 5%–13% in persons aged 70% and 11%–50% in those over 80 [1]. In China, sarcopenia affects 10.6%–15% of adults aged ≥ 60 years [7, 15], while a meta-analysis of hospitalized Chinese older adults found higher prevalence's of 29.7% in men and 23.0% in women [16]. A most recent meta-analysis reported that the pooled prevalence of sarcopenia in patients with T2DM was 18% [17]. Our findings showed that 17.41% of T2DM patients had low muscle mass (20.28% in males and 14.20% in women). Our result was consistent with the Chinese epidemiological survey that the prevalence of low muscle mass in males is higher than in females [18]. This gender disparity suggests more muscle loss in middle-aged and elderly men than women.

Methodological differences also contribute to prevalence variations. The prevalence in older men varied depending on the method used to assess muscle mass. Compared with the bioelectrical impedance analysis, the risk of prevalence was higher through DXA method [16]. However, no such difference was found in older females. Disorders of glucose metabolism increase the risk of muscle mass loss. The mechanisms of sarcopenia in T2DM patients are still complex and unclear: increased oxidative stress and reactive oxygen, insufficient protein uptake and synthesis and increased decomposition and consumption, changes in hormone levels, decreased insulin-like growth factor-1, and diabetes chronic complications [17]. Age remains a primary risk factor, with sarcopenia prevalence rising markedly in older populations along with age-related muscle loss [7]. According to Du et al. [19], sarcopenia was linked to blood lipid levels and BMI. Some studies have shown that BMI is negatively associated with sarcopenia [5]. Likewise, we discovered that patients with decreased muscle mass had lower BMIs and were older. These results imply that weight might act as a buffer against a lack of muscular mass.

Mg is essential for both metabolic signaling pathways and basic cellular functions. It is a necessary mineral to maintain the health and functionality of muscles [20]. Mg is linked to performance, strength, and muscular mass [21]. Notably, intracellular Mg deficiency is a contributor to sarcopenia, promoting mitochondrial oxidative stress and dysfunction in skeletal muscle [22, 23]. Previous research has primarily focused on the association between Mg intake and muscle mass. Mg supplementation enhances physical performance and promotes muscle strength in older adults [12, 24, 25]. Conversely, inadequate Mg intake contributes to the loss of muscle mass and reduced grip strength in older individuals [11]. Supporting this, cohort research indicates that the highest tertiles of Mg consumption are associated with the lowest likelihood of sarcopenia [24]. Furthermore, a systematic review and meta-analysis concluded that people with an Mg deficiency, such as older people, may benefit from Mg supplementation, but those with an adequate Mg status derive no significant advantage [26].

However, the associations between skeletal muscle mass and serum Mg concentration remain less clear. An epidemiological study demonstrated a significant positive correlation between serum Mg levels and muscle function in older adults [27]. Nevertheless, despite sarcopenia patients were found to have significantly lower Mg intakes compared to non-sarcopenia patients, this difference was not reflected in their serum Mg levels [28]. To the best of our knowledge, this is the first study to investigate the relationship between low muscle mass and serum Mg levels in Chinese patients with T2DM. Our findings revealed a negative correlation between SMI and serum Mg concentration. Furthermore, high serum Mg levels were associated with an increased risk of low muscle mass in women. Our previous preprint manuscript also confirmed the same conclusion that when the Mg levels were divided into quartiles, the risk of low muscle mass in the highest quartile group was significantly higher than that in the lowest quartile group in the preprint [29]. These results appear to contradict traditional perspectives. The possible explanations are as follows. Firstly, serum Mg constitutes only a small fraction (approximately 0.3%) of total body Mg, with the vast majority bound in tissues and playing crucial roles in various enzymatic functions [30]. Approximately 27% of the body's Mg^2+^ is stored in the skeletal muscle Mg pool. Serum Mg levels are tightly regulated within a narrow reference interval of 0.75–0.96 mmol/L [31]. In our study, 99.44% of the participants had serum Mg levels that were still within the normal laboratory reference range. However, individuals with normal serum Mg concentrations may still exhibit significant total body Mg deficiency, indicating that serum Mg concentration is not a reliable indicator of overall Mg status. Secondly, in healthy individuals, serum Mg levels are tightly regulated through homeostatic mechanisms and remain remarkably stable [32]. A dynamic equilibrium between intake, intestinal absorption, renal reabsorption, and bone storage is necessary for Mg homeostasis [33]. For instance, inadequate dietary Mg intake can cause intestinal Mg absorption to rise from 30% to 50%–80% to 90%. Approximately 90%–95% of the daily filtered Mg in the kidney is resorbed. Serum Mg homeostasis is maintained by all three mechanisms between 0.65 and 1.05 mmol/L [34]. Despite serum Mg measurement being the most commonly used method, it does not always accurately reflect intracellular Mg concentrations. It may fail to detect depleted Mg stores or persistent subclinical Mg deficiency. Intracellular Mg levels may differ significantly from serum Mg levels [35]. Total serum Mg concentrations do not necessarily reflect the Mg status of the intracellular compartment; ionized Mg depletion may occur even when serum Mg levels remain within the normal range [36]. According to a study on the relationship between age and intracellular Mg content using 31P-nuclear magnetic resonance (NMR) spectroscopy, older patients' intracellular Mg levels continuously decreased with age, although total serum Mg did not change significantly with age [37]. These findings may partially explain the inconsistencies with previous conclusions. Furthermore, we hypothesize that the Mg transport system may be dysfunctional in T2DM patients, potentially leading to intracellular Mg deficiency despite normal or even elevated serum Mg levels.

The present study contains several limitations. First, the sample size was small. Second, the conclusions' strength was constrained by the data's cross-sectional character. Future longitudinal research is required to confirm the causal link. Finally, serum Mg concentrations may not be the most reliable indicator for determining Mg status.

## 5. Conclusion

In conclusion, our study reveals novel evidence establishing an association between serum Mg concentration and low muscle mass in patients with T2DM. Specifically, among female patients, there is a linear trend and a significant negative correlation between Mg and low muscle mass. However, further investigations are warranted to clarify the precise relationships between the serum Mg concentration and skeletal muscle mass and between the serum Mg concentration and the intracellular Mg concentration. It is necessary to explore the characteristics and mechanism of serum and intracellular Mg transport in patients with T2DM.

## Figures and Tables

**Table 1:** The differences between T2DM patients with and without low muscle mass.

Characteristics	Total (*n* = 1074)	Male (*n* = 567)			Female (*n* = 507)		
		Normal muscle mass (*n* = 452)	Low muscle mass (*n* = 115)	*p* value	Normal muscle mass (*n* = 435)	Low muscle mass (*n* = 72)	*p* value
Age (years)	58.65 ± 12.16	54.69 ± 12.00	60.36 ± 13.51	< 0.001	61.18 ± 10.77	65.47 ± 11.28	0.002
Mg (mg/dL)	2.05 ± 0.17	2.03 ± 0.15	2.08 ± 0.19	0.004	2.05 ± 0.18	2.12 ± 0.20	0.002
SMI (kg/m^2^)	7.07 ± 1.23	8.11 ± 0.83	6.45 ± 0.48	< 0.001	6.48 ± 0.85	5.07 ± 0.33	< 0.001
BMI (kg/m^2^)	26.63 ± 4.43	27.46 ± 3.88	23.70 ± 4.90	< 0.001	27.25 ± 4.34	22.42 ± 2.98	< 0.001
SBP (mmHg)	141.64 ± 20.45	140.68 ± 19.46	137.37 ± 21.40	0.112	143.47 ± 20.69	143.07 ± 21.95	0.879
DBP (mmHg)	78.90 ± 12.99	82.49 ± 12.85	76.62 ± 12.58	< 0.001	76.59 ± 12.71	74.83 ± 11.07	0.271
Duration (years)	8.53 ± 6.34	8.1 ± 6.71	8.96 ± 5.90	0.207	8.76 ± 5.99	9.17 ± 6.73	0.599
TC (mmol/L)	4.60 ± 1.23	4.52 ± 1.17	4.51 ± 1.06	0.894	4.70 ± 1.36	4.60 ± 1.16	0.548
TG (mmol/L)	1.97 ± 1.79	2.31 ± 2.27	1.43 ± 0.81	< 0.001	1.85 ± 1.40	1.44 ± 0.79	0.016
LDL (mmol/L)	2.99 ± 0.93	2.98 ± 0.92	2.99 ± 0.92	0.936	3.00 ± 0.94	2.89 ± 0.98	0.370
HDL (mmol/L)	1.23 ± 0.34	1.14 ± 0.27	1.24 ± 0.35	0.006	1.30 ± 0.37	1.36 ± 0.35	0.192
FPG (mmol/L)	7.93 ± 2.89	8.20 ± 2.78	8.02 ± 3.19	0.552	7.74 ± 2.90	7.04 ± 2.76	0.055
HbA1c (%)	8.43 ± 2.08	8.45 ± 2.09	8.96 ± 2.18	0.025	8.32 ± 2.02	8.15 ± 2.13	0.545
Ca (mmol/L)	2.30 ± 0.12	2.29 ± 0.11	2.28 ± 0.13	0.277	2.30 ± 0.12	2.30 ± 0.13	0.888
Phosphorus (mmol/L)	1.21 ± 0.19	1.20 ± 0.19	1.15 ± 0.20	0.008	1.25 ± 0.18	1.23 ± 0.23	0.459
Scr (umol/L)	64.35 ± 29.07	69.96 ± 20.18	74.30 ± 66.19	0.490	56.59 ± 15.54	60.54 ± 31.08	0.294
BUN (mmol/L)	5.44 ± 1.87	5.57 ± 1.71	5.87 ± 2.69	0.258	5.20 ± 1.60	5.43 ± 2.51	0.305
Smoking				0.563			0.995
Yes (n)	315 (29.4%)	243 (54%)	65 (57%)		6 (1.4%)	1 (1.4%)	
No (n)	756 (70.6%)	207 (46%)	49 (43%)		429 (98.6%)	71 (98.6%)	
Alcohol				0.789			0.480
Yes (n)	254 (23.7%)	199 (44.2%)	52 (45.6%)		3 (0.7%)	0	
No (n)	817 (76.3%)	251 (55.8%)	62 (54.4%)		432 (99.3%)	72 (100%)	
Metformin (*n*, %)	741 (69.5%)	308 (68.8%)	74 (64.9%)	0.433	316 (73.1%)	43 (59.7%)	0.020
Acarbose (*n*, %)	389 (36.5%)	152 (33.9%)	40 (35.1%)	0.816	173 (40%)	24 (33.3%)	0.286
Sulfonylureas (*n*, %),	310 (29.1%)	114 (25.4%)	43 (37.7%)	0.009	132 (30.6%)	21 (29.2%)	0.812
DPP-IV inhibitor (*n*, %)	209 (19.6%)	84 (18.8%)	31 (27.2%)	0.046	81 (18.7%)	13 (18.1%)	0.895
Insulin (*n*, %)	411 (38.6%)	168 (37.7%)	42 (36.8%)	0.871	176 (40.6%)	25 (34.7%)	0.342
Statin (*n*, %)	184 (17.3%)	78 (17.4%)	23 (20.2%)	0.499	73 (16.9%)	10 (13.9%)	0.524

*Note:* Mg: magnesium; HbA1c: glycosylated hemoglobin; Scr: serum creatinine; Ca: calcium; P: phosphorus.

Abbreviations: BMI, body mass index; BUN, blood urea nitrogen; DBP, diastolic blood pressure; FPG, fasting plasma glucose; HDL-C, high-density lipoprotein cholesterol; LDL-C, low-density lipoprotein cholesterol; SBP, systolic blood pressure; SMI, skeletal muscle mass index; TC, total cholesterol; TG, triglyceride.

**Table 2:** The association between SMI and clinical characteristics in male subjects.

Characteristics	Total (*n* = 567)	Simple regression analysis		Multiple regression analysis	
		*β* (95% CI)	*p* value	*β* (95% CI)	*p* value
Age (years)	55.84 ± 12.52	−0.025 (−0.032, −0.019)	< 0.001	−0.019 (−0.026, −0.011)	< 0.001
Mg (mg/dL)	2.04 ± 0.16	−0.770 (−1.286, −0.255)	0.003	−0.416 (−0.888, 0.056)	0.084
SBP (mmHg)	140.01 ± 19.89	0.006 (0.002, 0.011)	0.003	0.006 (0.002, 0.010)	0.007
DBP (mmHg)	81.30 ± 13.00	0.019 (0.013, 0.025)	< 0.001	0.008 (0.002, 0.015)	0.014
Duration (years)	8.45 ± 6.51	−0.020 (−0.033, −0.007)	0.002	−0.001 (−0.014, 0.011)	0.840
TC (mmol/L)	4.52 ± 1.14	0.042 (−0.032, 0.115)	0.267		
TG (mmol/L)	2.13 ± 2.09	0.087 (0.047, 0.127)	< 0.001	0.038 (0.001, 0.075)	0.043
LDL (mmol/L)	2.99 ± 0.92	0.058 (−0.034, 0.150)	0.218		
HDL (mmol/L)	1.16 ± 0.29	−0612 (−0.897, −0.327).	< 0.001	−0.576 (−0842, −0.311)	< 0.001
FPG (mmol/L)	8, 16 ± 2.87	0.024 (−0.005, 0.054)	0.101		
HbA1c (%)	8.55 ± 2.12	−0.036 (−0.077, 0.006)	0.091		
Ca (mmol/L)	2.29 ± 0.12	0.805 (0.085, 1.526)	0.029	0.483 (−0.190, 1.156)	0.159
P (mmol/L)	1.19 ± 0.19	0.868 (0.429, 1.307)	< 0.001	0.337 (−0.092, 0.766)	0.123
Scr (umol/L)	70.84 ± 34.77	−0.001 (−0.003, 0.001)	0.445		
BUN (mmol/L)	5.63 ± 1.95	−0.046 (−0.090, −0.003)	0.035	−0.007 (−0.048, 0.033)	0.717
Smoking	308 (54.3%)	0.009 (−0.160, 0.178)	0.916		
Alcohol	251 (44.3%)	0.086 (−0.083, 0.255)	0.319		
Metformin	382 (67.4%)	0.042 (−0.137, 0.221)	0.647		
Acarbose	192 (33.9%)	−0.023 (−0.200, 0.153)	0.794		
Sulfonylureas	157 (27.7%)	−0.213 (−0.399, −0.027)	0.025	−0.120 (−0.292, 0.052)	0.171
DPP-IV inhibitor	115 (20.3%)	−0.260 (−0.467, −0.054)	0.013	−0.194 (−0.378, −0.010)	0.039
Insulin	210 (37.0%)	−0.007 (−0.181, 0.166)	0.934		
Statin	101 (17.8%)	0.023 (−0.195, 0.241)	0.207		

*Note:* Mg: magnesium; HbA1c: glycosylated hemoglobin; Scr: serum creatinine; Ca: calcium; P: phosphorus.

Abbreviations: BMI, body mass index; BUN, blood urea nitrogen; DBP, diastolic blood pressure; FPG, fasting plasma glucose; HDL-C, high-density lipoprotein cholesterol; LDL-C, low-density lipoprotein cholesterol; SBP, systolic blood pressure; TC, total cholesterol; TG, triglyceride.

**Table 3:** The association between SMI and clinical characteristics in female subjects.

Characteristics	Total (*n* = 507)	Simple regression analysis		Multiple regression analysis	
		*β* (95% CI)	*p* value	*β* (95% CI)	*p* value
Age (years)	61.79 ± 10.93	−0.017 (−0.025, −0.010)	< 0.001	−0.014 (−0.022, −0.007)	< 0.001
Mg (mg/dL)	2.06 ± 0.18	−0.618 (−1.061, −0.175)	0.006	−0.438 (−0.872, −0.003)	0.048
SBP (mmHg)	143.42 ± 20.85	0.003 (−0.001, 0.007)	0.113		
DBP (mmHg)	76.34 ± 12.50	0.012 (0.005, 0.018)	0.001	0.009 (0.003, 0.015)	0.007
Duration (years)	9.07 ± 5.99	0.000 (−0.013, 0.013)	0.980		
TC (mmol/L)	4.69 ± 1.33	−0.006 (−0.068, 0.056)	0.848		
TG (mmol/L)	1.79 ± 1.34	0.052 (−0.009, 0.114)	0.093		
LDL (mmol/L)	2.98 ± 0.94	0.000 (−0.087, 0.087)	0.997		
HDL (mmol/L)	1.30 ± 0.37	−0.228 (−0.452, −0.004)	0.046	−0.193 (−0.411, 0.025)	0.083
FPG (mmol/L)	7.64 ± 2.89	0.003 (−0.026, 0.031)	0.858		
HbA1c (%)	8.30 ± 2.03	−0.008 (−0.050, 0.033)	0.696		
Ca (mmol/L)	2.30 ± 0.12	0.232 (−0.466, 0.929)	0.514		
P (mmol/L)	1.25 ± 0.19	0.642 (0.207, 1.077)	0.004	0.555 (0.130, 0.979)	0.011
Scr (umol/L)	57.15 ± 18.56	0.000 (−0.004, 0.005)	0.879		
BUN (mmol/L)	5.24 ± 1.76	−0.006 (−0.053, 0.041)	0.804		
Smoking	7 (1.4%)	0.335 (−0.369, 1.039)	0.350		
Alcohol	3 (0.6%)	0.100 (−0.972, 1.172)	0.855		
Metformin	359 (70.8%)	0.152 (−0.026, 0.331)	0.094		
Acarbose	197 (38.9%)	0.019 (−0.147, 0.185)	0.820		
Sulfonylureas	153 (30.2%)	0.089 (−0.087, 0.265)	0.320		
DPP-IV inhibitor	94 (18.5%)	−0.036 (−0.244, 0.171)	0.732		
Insulin	201 (39.6%)	0.120 (−0.045, 0.284))	0.155		
Statin	83 (16.4%)	0.039 (−0.179, 0.257)	0.725		

*Note:* Mg: magnesium; HbA1c: glycosylated hemoglobin; Scr: serum creatinine; Ca: calcium; P: phosphorus.

Abbreviations: BMI, body mass index; BUN, blood urea nitrogen; DBP, diastolic blood pressure; FPG, fasting plasma glucose; HDL-C, high-density lipoprotein cholesterol; LDL-C, low-density lipoprotein cholesterol; SBP, systolic blood pressure; TC, total cholesterol; TG, triglyceride.

**Table 4:** The association between Mg and other clinical characteristics in male subjects.

Characteristics	Total (*n* = 567)	Simple regression analysis		Multiple regression analysis	
		*β* (95% CI)	*p* value	*β* (95% CI)	*p* value
Age (years)	55.84 ± 12.52	0.002 (0.001, 0.003)	< 0.001	0.001 (0.000, 0.003)	0.011
BMI (kg/m^2^)	26.70 ± 4.37	−0.004 (−0.007, −0.001)	0.009	−0.003 (−0.006, 0.000)	0.066
SBP (mmHg)	140.01 ± 19.89	0.000 (0.000, 0.001)	0.290		
DBP (mmHg)	81.30 ± 13.00	−0.001 (−0.002, 0.000)	0.151		
Duration (years)	8.45 ± 6.51	0.000 (−0.002, 0.002)	0.975		
TC (mmol/L)	4.52 ± 1.14	0.013 (0.001, 0.024)	0.034	0.021 (0.009, 0.033)	0.001
TG (mmol/L)	2.13 ± 2.09	−0.002 (−0.008, 0.004)	0.532		
LDL (mmol/L)	2.99 ± 0.92	0.014 (−0.001, 0.028)	0.068		
HDL (mmol/L)	1.16 ± 0.29	0.046 (0.000, 0.091)	0.051		
FPG (mmol/L)	8,16 ± 2.87	−0.011 (−0.016, −0.007)	< 0.001	−0.008 (−0.013, −0.002)	0.005
HbA1c (%)	8.55 ± 2.12	−0.012 (−0.018, −0.005)	< 0.001	−0.006 (−0.013, 0.001)	0.096
Ca (mmol/L)	2.29 ± 0.12	0.022 (−0.093, 0.138)	0.706		
P (mmol/L)	1.19 ± 0.19	0.006 (−0.065, 0.077)	0.875		
Scr (umol/L)	70.84 ± 34.77	0.002 (0.001, 0.002)	< 0.001	0.001 (0.001, 0.002)	< 0.001
BUN (mmol/L)	5.63 ± 1.95	0.017 (0.010, 0.024)	< 0.001	0.002 (−0.007, 0.011)	0.643
Smoking	308 (54.3%)	−0.012 (−0.039, 0.015)	0.385		
Alcohol	251 (44.3%)	−0.018 (−0.046, 0.009)	0.180		
Metformin	382 (67.4%)	−0.036 (−0.065, −0.008)	0.013	−0.037 (−0.064, −0.009)	0.010
Acarbose	192 (33.9%)	−0.017 (−0.045, 0.012)	0.253		
Sulfonylureas	157 (27.7%)	0.002 (−0.028, 0.032)	0.877		
DPP-IV inhibitor	115 (20.3%)	−0.005 (−0.038, 0.029)	0.785		
Insulin	210 (37.0%)	−0.004 (−0.032, 0.024)	0.799		
Statin	101 (17.8%)	0.023 (−0.013, 0.058)	0.206		

*Note:* HbA1c: glycosylated hemoglobin; Scr: serum creatinine; Ca: calcium; P: phosphorus.

Abbreviations: BMI, body mass index; BUN, blood urea nitrogen; DBP, diastolic blood pressure; FPG, fasting plasma glucose; HDL-C, high-density lipoprotein cholesterol; LDL-C, low-density lipoprotein cholesterol; SBP, systolic blood pressure; TC, total cholesterol; TG, triglyceride.

**Table 5:** The association between Mg and other clinical characteristics in female subjects.

Characteristics	Total (*n* = 507)	Simple regression analysis		Multiple regression analysis	
		*β* (95% CI)	*p* value	*β* (95% CI)	*p* value
Age (years)	61.79 ± 10.84	0.002 (0.001, 0.004)	0.002	0.002 (0.001, 0.004)	0.008
BMI (kg/m^2^)	26.56 ± 4.50	−0.006 (−0.010, −0.003)	0.001	−0.006 (−0.009, −0.002)	0.001
SBP (mmHg)	143.42 ± 20.85	0.000 (−0.001, 0.001)	0.979		
DBP (mmHg)	76.34 ± 12.50	0.000 (−0.001, 0.001)	0.764		
Duration (years)	9.07 ± 5.99	−0.004 (−0.006, −0.001)	0.005	−0.001 (−0.004, 0.001)	0.328
TC (mmol/L)	4.69 ± 1.33	−0.001 (−0.013, 0.011)	0.842		
TG (mmol/L)	1.79 ± 1.34	−0.017 (−0.029, −0.005)	0.004	0.002 (−0.011, 0.014)	0.3785
LDL (mmol/L)	2.98 ± 0.94	−0.003 (−0.020, 0.014)	0.701		
HDL (mmol/L)	1.30 ± 0.37	0.022 (−0.022, 0.066)	0.328		
FPG (mmol/L)	7.64 ± 2.89	−0.016 (−0.021, −0.011)	< 0.001	−0.010 (−0.016, −0.003)	0.005
HbA1c (%)	8.30 ± 2.03	−0.021 (−0.029, −0.013)	< 0.001	−0.007 (−0.017, 0.003)	0.155
Ca (mmol/L)	2.30 ± 0.12	−0.046 (−0.183, 0.090)	0.506		
P (mmol/L)	1.25 ± 0.19	−0.075 (−0.161, 0.011)	0.087		
Scr (umol/L)	57.15 ± 18.56	0.002 (0.001, 0.002)	< 0.001	0.002 (0.001, 0.002)	< 0.001
BUN (mmol/L)	5.24 ± 1.76	0.008 (−0.001, 0.017)	0.078		
Smoking	7 (1.4%)	−0.152 (−0.289, −0.014)	0.031	−0.090 (−0.233, 0.053)	0.216
Alcohol	3 (0.6%)	−0.218 (−0.427, −0.009)	0.041	−0.097 (−0.315, 0.120)	0.379
Metformin	359 (70.8%)	−0.081 (−0.116, −0.046)	< 0.001	−0.051 (−0.088, −0.014)	0.007
Acarbose	197 (38.9%)	−0.043 (−0.076, −0.010)	0.011	−0.009 (−0.042, 0.023)	0.577
Sulfonylureas	153 (30.2%)	−0.052 (−0.087, −0.017)	0.004	−0.035 (−0.069, 0.000)	0.049
DPP-IV inhibitor	94 (18.5%)	−0.013 (−0.054, 0.029)	0.543		
Insulin	201 (39.6%)	−0.078 (−0.111, −0.046)	< 0.001	−0.069 (−0.103, −0.034)	< 0.001
Statin	83 (16.4%)	0.026 (−0.018, 0.070)	0.243		

*Note:* HbA1c: glycosylated hemoglobin; Scr: serum creatinine; Ca: calcium; P: phosphorus.

Abbreviations: BMI, body mass index; BUN, blood urea nitrogen; DBP, diastolic blood pressure; FPG, fasting plasma glucose; HDL-C, high-density lipoprotein cholesterol; LDL-C, low-density lipoprotein cholesterol; SBP, systolic blood pressure; TC, total cholesterol; TG, triglyceride.

**Table 6:** The association between Mg and low muscle mass in male subjects.

Variables	Low muscle mass/*N* (%)	Unadjusted Model 1		Model 2		Model 3	
		OR (95% CI)	*p* value	OR (95% CI)	*p* value	OR (95% CI)	*p* value
Mg (mg/dL, median[range])							
T1 (1.87[< 1.96])	32/171 (18.71%)	1.000 (Reference)		1.000 (Reference)		1.000 (Reference)	
T2 (2.04[1.96–2.11])	33/191 (17.28%)	0.907 (0.530–1.552)	0.722	0.829 (0.480–1.432)	0.501	0.734 (0.409–1.316)	0.299
T3 (2.16[≥ 2.11])	50/205 (24.39%)	1.401 (0.850–2.309)	0.185	1.196 (0.716–1.996)	0.494	0.988 (0.556–1.757)	0.969
*P* for trend			0.154		0.403		0.971

*Note:* Model 1 was a crude model; Model 2 was adjusted for age; Model 3 was adjusted for age, duration of T2DM, SBP, DBP, TG, TC, HDL-C, LDL-C, FPG, BUN, Scr, P, and Ca.

Abbreviations: CI, confidence interval; OR, odds ratio; T, tertile.

**Table 7:** The association between low muscle mass and Mg in female subjects.

Variables	Low muscle mass/*N* (%)	Unadjusted Model 1		Model 2		Model 3	
		OR (95% CI)	*p* value	OR (95% CI)	*p* value	OR (95% CI)	*p* value
Mg (mg/dL, median[range])							
T1 (1.87[< 1.96])	13/155 (8.39%)	1.000 (Reference)		1.000 (Reference)		1.000 (Reference)	
T2 (2.04[1.96–2.12])	22/171 (12.87%)	1.613 (0.783–3.324)	0.195	1.479 (0.713–3.066)	0.293	1.321 (0.626–2.790)	0.465
T3 (2.24[≥ 2.12])	37/181 (20.44%)	2.807 (1.432–5.502)	0.003	2.481 (1.254–4.905)	0.009	2.071 (1.011–4.243)	0.047
*P* for trend			0.001		0.006		0.039

*Note:* Model 1 was a crude model; Model 2 was adjusted for age; Model 3 was adjusted for age, duration of T2DM, SBP, DBP, TG, TC, HDL-C, LDL-C, FPG, BUN, Scr, P, and Ca.

Abbreviations: CI, confidence interval; OR, odds ratio; T: tertile.

## Data Availability

Data are available from the corresponding author upon reasonable request.

## General Statement

During the previous submission process, there was a preprint [Lili Pei, Qiao Yang, Yuantao Liu, and Wenchao Hu (2025). Serum Magnesium is correlated with Muscle Mass in Patients with Type 2 Diabetes Mellitus. Preprint, researchsquare.com] https://doi.org/10.21203/rs.3.rs-5871854/v1. There are many differences between the preprint and this manuscript. Based on the same research population, the results in the statistical description section are consistent. In addition, we made many revisions to the manuscript, such as modifying the statistical methods, tables, figures, and the discussion section. We determined that this manuscript is the final version.
